# Cell Membrane-Coated Halloysite Nanotubes for Target-Specific Nanocarrier for Cancer Phototherapy

**DOI:** 10.3390/molecules26154483

**Published:** 2021-07-25

**Authors:** Cuiying Tan, Jingqi Zheng, Yue Feng, Mingxian Liu

**Affiliations:** Department of Materials Science and Engineering, Jinan University, Guangzhou 510632, China; tancy939690@163.com (C.T.); 373642635@163.com (J.Z.); yuef_1016@163.com (Y.F.)

**Keywords:** nanotubes, cancer, antibody, cell membrane, photothermal

## Abstract

Naturally-occurring halloysite nanotubes (HNTs) have many advantages for constructing target-specific delivery of phototherapeutic agents. Here, HNTs were labeled with fluorescein isothiocyanate (FITC) and loaded with the type-II photosensitizer indocyanine green (ICG) for phototherapy. HNTs-FITC-ICG was structurally stable due to presence of HNTs as the nanocarrier and protective agent. The nanocarrier was further wrapped with red blood cell membrane (RBCM) to enhance the biocompatibility. The HNTs-FITC-ICG-RBCM nanocarrier show high cytocompatibility and hemocompatibility. Due to the photothermal effect of ICG, a significant temperature rising was achieved by irradiation of the nanocarrier using 808 nm laser. The photothermal temperature rising was used to kill the cancer cells effectively. The HNTs-FITC-ICG-RBCM nanocarrier was further linked with anti-EpCAM to endow it with targeting therapy performance against breast cancer, and the anti-EpCAM-conjugated nanocarrier exhibited significantly tumor-specific accumulation. The RBCM-coated and biocompatible HNTs nanocarrier is a promising candidate for target-specific therapy of cancer.

## 1. Introduction

Breast cancer is the highest incidence rate of cancer in females globally (11.7% of total cases), and is one of the leading causes of death among women [1]. The American Cancer Society has reported 316,700 estimated new cases breast cancer and 41,760 breast cancer-related death among US women in 2019 [2], and there is an increase trend in breast cancer incidence rates (about 0.5% per year) [3]. Currently, the clinical treatment for breast cancer mainly includes surgery, radiation therapy and chemotherapy (usually including targeted therapy and immunotherapy) [4,5]. However, there is still a series of critical challenges for treatment of the breast cancer, such as damage to normal tissue, recurrence of cancer and occurrence of drug resistance [6]. To overcome the defect of conventional treatments, nanoparticle-based chemotherapy has been explored as an effective and innovative anti-cancer strategy [7,8]. Functional nanoparticles can be used as therapeutic agents or drug carriers. Compared with traditional chemical drugs, nanomedicines have many advantages such as improved in vivo bioavailability, favorable targeting and controllable drug release ability. So far, a variety of organic nanoparticles have shown their highly efficiency in the treatment of breast cancer with low toxicity [9], including liposomes [10,11], polymer micelles [12,13] and dendrimers [14,15]. In addition, inorganic nanoparticles have attracted considerable attention due to their nanoscale size, good stability and ease of synthesis and modification [16]. Compared with organic nanoparticles which usually use in vivo stimulation to achieve controlled drug release, inorganic nanoparticles can use external stimuli such as light, magnetic field irradiation and ultrasound to kill cancer cells directly, which has more advantages on the temporal and spatial control of cancer therapy [17]. 

As innocuous and available inorganic nanomaterials, halloysite nanotubes (HNTs) have considerable advantages as a drug carrier for cancer therapy. HNTs are natural aluminosilicate clay with a hollow tubular structure, and their chemical formula is Al_2_(OH)_4_Si_2_O_5_·*n*H_2_O. The nanotubes range in length from 200 nm to 1500 nm, while the outer and inner diameters are 40–70 nm and 10–30 nm, respectively [18]. Structurally, HNTs are multilayer tubular structure with chemically different internal (-Al-OH groups) and external surfaces (Si–O-Si group) [19]. Thanks to their special chemical composition, HNTs show different reactivity on the inner and outer surface, which enables them rich functionalization possibilities for various applications [20]. Moreover, in virtue of the good biocompatibility, high absorbability, large volume of cavity and low cost, HNTs possess broad application potentials in the field of biomedicine [18,21]. In recent years, HNTs have demonstrated superior performance in controlling release of herbicide [22,23], antimicrobial [24], protein [25] and gene [26]. Due to its high loading capacity and biocompatibility, HNTs are also promising candidates in intracellular drug delivery, which have been applied as drug carrier for cancer therapy [27]. A variety of chemical reagents were explored to modify HNTs to improve their anticancer efficiency. For example, enhanced drug loading efficiency and anticancer activity in tumor cell were discovered in chitosan modified HNTs [28]. In addition, folic acid grafted HNTs were able to employ as active cancer targeted platform to deliver doxorubicin drug [29].

Functionalized HNTs have been applied as carriers of photosensitizer drugs for cancer therapy [30,31]. To enhance anti-cancer efficacy and reduce side effects, phototherapy as a new treatment method to kill tumor cells through reactive oxygen species and thermal effects has received considerable attention [32]. Malignant cells in vivo appeared to be destroyed by hyperthermia in the temperature range of 41–43 °C [33]. In contrast, normal cells are not as sensitive to temperature as malignant cells. However, the nanoparticles employed in phototherapy are always able to trigger an immune response, leading to a shortened circulation time and limited therapeutic effectiveness [34]. Owning to the abundant proteins on the cell surface, cell membranes can be applied to camouflage nanoparticles to avoid the immune system [35]. Thus, cell-membrane coating has emerged as an effective solution to the dilemma of nanoparticles. Among the used cell for coating nanoparticles, red blood cells (RBCs) are the excellent candidate due to their inherent biocompatibility and low immunogenicity [36,37]. It has been found that nanoparticles coated RBC membrane (RBCM) possessed outstanding physiological stability, prolonged blood circulation and enhanced tumor-homing capacity [38,39,40].

**Scheme 1:**
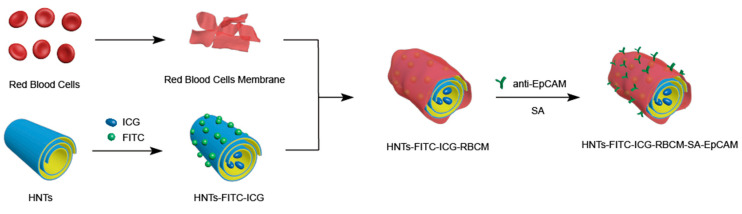
Synthesis process of HNTs-FITC-ICG-RBCM-SA-EpCAM.

In this work, a HNTs-based multifunctional nanoparticle is designed for tumor targeting and phototherapy in breast cancer therapy. Fluorescein isothiocyanate (FITC) was first adsorbed on the surfaces of HNTs, and indocyanine green (ICG) was then loaded as the photothermal agent into the lumen. To enhance the biocompatibility of the nanoparticle, RBCM coating was then wrapped on the surface of the HNTs-FITC-ICG. Finally, anti-EpCAM was conjugated with HNTs-FITC-ICG-RBCM under the help of streptavidin (SA) to improve the specific uptake of breast cancer cells (Scheme 1). Physicochemical properties of the nanoparticles were investigated by various characterization methods. Moreover, cell counting Kit-8 (CCK-8) and flow cytometric analysis were employed to study the photothermal and antitumor effect of this nanoplatform. From these results, the nanoparticles exhibited improved anticancer efficiency while showed lower toxicity to normal tissues. The novel phototherapy nanoplatform of HNTs-FITC-ICG-RBCM-SA-EpCAM has shown the potential application value in the treatment of breast cancer. 

## 2. Materials and Methods

### 2.1. Materials

HNTs were provided by Guangzhou Runwo Materials Technology Co., Ltd., China and used without further purification. The elemental composition of used HNTs by X-ray fluorescence (XRF) was determined as follows (wt%): SiO_2_, 58.91; Al_2_O_3_, 40.41; Fe_2_O_3_, 0.275; P_2_O_5_, 0.138; TiO_2_, 0.071. The weight loss of HNTs was 19.20%, which was mainly occurred in the temperature range of 400–500 °C. Fluorescein isothiocyanate (FITC) (analytical grade) was purchased from Shanghai Yeasen Biotechnology Co., Ltd., Shanghai, China. Indocyanine green (ICG) and streptavidin (SA) were purchased from Sigma-Aldrich (St. Louis, MO, US). Anti-EpCAM aptamer was purchased from Sangon Biotech, Shanghai. Acridine orange (AO) and ethidium bromide (EB) were purchased from Solarbio Science and Technology Co., Ltd., Beijing, China. Ultrapure water was obtained from Milli-Q water system. All the other chemicals were analytically graded and used directly without further purification.

### 2.2. Preparation of HNTs-FITC-ICG

#### 2.2.1. Preparation of FITC-Labeled HNT

HNTs-FITC were firstly prepared by mixing HNTs with FITC in ultrapure water. The HNTs powder was dissolved in 100 mL ultrapure water under magnetic stirring at room temperature for 30 min, and dispersion was then ultrasonically treated in an ice bath for 30 min. After adding of the FITC to the dispersion, the dispersion was homogenized through magnetic stirring for 24 h in the absence of light. HNTs-FITC were then obtained by washing the product using water and anhydrous ethanol with a centrifuge (YNX-4000, Thermo Fisher Scientific Ltd., Waltham, MA, USA). The prepared HNTs-FITC were dried by freezer dryer (Scientz-18ND, Ningbo Scientz Biotechnology Ltd., Zhejiang, China), and the dried product was stored in a dry and dark environment.

#### 2.2.2. Loading ICG into HNTs-FITC (HNTs-FITC-ICG) 

HNTs-FITC were dispersed in water and sterilized in a microwave oven. In addition, the ICG was dissolved in water and filtered twice with 0.22 μm filter, obtaining sterile ICG aqueous solution. Then, sterile HNTs-FITC was added to the ICG aqueous solution, stirred for one day, and then the HNTs-FITC-ICG were prepared. The obtained HNTs-FITC-ICG were washed three times under sterile conditions.

### 2.3. Preparation of HNTs-FITC-ICG-RBCM-SA-EpCAM

#### 2.3.1. Extraction of RBCM

5 mL of whole blood was extracted from healthy rabbit. Proper amount of normal saline was applied to wash the whole blood for 3 times by centrifugation (3000 rpm, 10 min). Then, swelling process was performed for 1 h to rupture red blood cells using 0.25× of normal saline. The obtained solution was centrifuged, discarding the supernatant, and red blood cell membranes were collected (RBCM). The obtained RBCM was dispersed into original whole blood volume (5 mL) with normal saline, which were diluted 10 times subsequently. To obtain a spare RBCM dispersion, the diluted solution was filtrated with 800 nm filters for 5 times followed by 450 nm filters for 5 times (It was easy to block if use 450 nm filter heads directly). The filtered solution was then centrifuged, and the misty and translucent solid was obtained which suggested the successful collection of RBCM.

#### 2.3.2. Preparation of RBCM-Coated HNTs-FITC-ICG (HNTs-FITC-ICG-RBCM)

HNTs-FITC-ICG and RBCM were mixed according to the ratio (HNTs-FITC-ICG (mass):RBCM (volume) = 1 mg:30 μL), and the dispersion was then ultrasonically treated to coat RBCM on HNTs-FITC-ICG in ice bath for 30 min. To remove the free RBCM, the supernatant was discarded after the sedimentation of the nanoparticles in dispersion which was then dispersed in fresh normal saline.

#### 2.3.3. Conjugation of Anti-EpCAM to HNTs-FITC-ICG-RBCM (HNTs-FITC-ICG-RBCM-SA-EpCAM)

The anti-EpCAM was conjugated with the modified HNTs according to previous study [41]. 10 μL of SA and anti-EpCAM were diluted 100 times with normal saline, respectively, to obtain 1 mL dilute solutions. Then, 0.5 g HNTs-FITC-ICG-RBCM were added into 1 mL SA dilute solution, and ultrasound was applied for 30 min in ice bath to mix them uniformly. Subsequently, 1 mL anti-EpCAM dilute solution was added and mixed under the same conditions as in the previous step. After sedimentation of the obtained solution, the supernatant was removed and the sediment was dispersed in fresh normal saline. The product was stable in aqueous dispersion for 3–4 h before gradually settling down. 

### 2.4. Characterization Methods

The size and morphology of the HNTs and HNTs-FITC-ICG-RBCM were characterized by transmission electron microscope (JEM-2100F, JEOL Ltd., Tokyo, Japan) at the accelerating voltage of 100 kV. To improve the contrast of TEM images, HNTs-FITC-ICG-RBCM was stained with uranyl acetate before analysis due to the low contrast of sample containing RBCM.

Zeta potential of HNTs-FITC-ICG-RBCM and other nanoparticles were measured by dynamic laser scattering apparatus (Malvern Instruments Ltd., Worcestershire, UK). All samples were dispersed with normal saline at a concentration of 0.05%.

To study the structure of FITC-labeled and ICG-loaded HNTs, FTIR spectra measurements were performed using a Thermo FTIR (Nicolet iS50, Thermo Fisher Scientific Ltd., Waltham, MA, USA) with the spectral range from 4000 to 400 cm^−1^.

### 2.5. Photothermal Effect of HNTs-FITC-ICG

To study the photothermal effect of HNTs-FITC-ICG, 1 mL of HNTs-FITC-ICG suspension with different concentrations (1, 2 and 3 mg/mL) was added to a 1.5 mL Eppendorf (EP) tube and was then irradiated under 808 nm laser using different power (1.2, 1.5 and 1.8 W/cm^2^). The temperature increases were recorded by infrared camera every 60 s while infrared photographs were taken every 2 min over the duration of 8 min. As a control group, water was irradiated by a 1.5 W/cm^2^ laser under the same conditions. To investigate the stability of HNTs-FITC-ICG, cyclical laser irradiation (808 nm, 1.5 W cm^−2^) was performed in 2 mg/mL HNTs-FITC-ICG suspension. Briefly, HNTs-FITC-ICG suspension was exposed to laser irradiation (808 nm, 1.5 W cm^−2^) for 2 min, then turned off the laser and cooled it naturally for 2 min. The cycle was repeated five times, and the infrared camera was also applied to record the temperature changes every minute while taking infrared images every 2 min. Photostability of HNTs-FITC-ICG suspension stored for different days were studied by measuring the photothermal effect of these suspensions under the same conditions stated above. Structural stability of HNTs-FITC-ICG was also evaluated by measuring the UV spectra of HNTs-FITC-ICG at different days (1, 3, 6 and 10 days) using an UV-Visible Spectrophotometer (UV-2550, Shimadzu Instrument Ltd., Suzhou, China).

### 2.6. Hemocompatibility

Hemolysis assay was carried out to investigate the hemocompatibility of HNTs, HNTs-FITC-ICG and HNTs-FITC-ICG-RBCM. To obtain red blood cells (RBCs) dispersion, 1 mL of fresh rabbit blood was mixed with 10 mL of normal saline and the mixture was treated with centrifugation (3000 rpm) for 5 min. After the centrifugal operation was repeated for 3 times, 2 mL normal saline was added to resuspend and disperse RBCs. Subsequently, 50 microliters of RBCs dispersion were mixed with 2 mL of normal saline. Afterward, 25 μL of HNTs, HNTs-FITC-ICG and HNTs-FITC-ICG-RBCM (0.59 mg/mL) were further added into the RBCs dispersion, respectively. These three mixed systems were incubated at 37 °C for 1 h. Then the obtained solutions were centrifuged at 1000 rpm for 5 min. To remove the influence of nanoparticles on absorbance, the supernatant was transferred and centrifuged at 15,000 rpm for 5 min. In this case, 100 microliters of the supernatant were taken into a 96-well plate and its absorbance was measured at 570 nm with a microplate analyzer. Ultrapure water was applied to treat the RBCs, and used as positive control group, which was corresponding to 100% hemolysis rate. Conversely, the RBCs were treated with PBS as the negative control group and the hemolysis was 0%. The hemolysis rate is calculated by the following formula:

(1) $$\mathrm{Hemolysis}\;\mathrm{ratio}\;\left(\%\right)=\frac{A_{sample}-A_{negative\;control}}{A_{positive\;control}-\;A_{negative\;control}}\;\times 100\%$$

Moreover, the morphology of RBCs treated with different nanoparticles were also observed using a universal optical microscope. The RBCs of the sample group in hemolysis assay after incubation with the sample were used to observe. In this case, 100 microliters of RBCs solutions were placed for 24 h before observation. 

### 2.7. Cell Culture

Human breast cancer cells (MCF-7) and human umbilical vein endothelial cells (HUVECs) were obtained from the Cell Bank of the China Academy of Sciences. Both cells were grown in Dulbecco’s modified Eagle’s medium (DMEM) within a humidified atmosphere containing 5% CO_2_ at 37 °C.

### 2.8. In Vitro Cytotoxicity Study

MCF-7 and HUVECs were seeded into the 96 well plates at a density of 1 × 10^4^ cells/well and incubated for 24 h. Then, different concentrations (50, 100, 200, 400, 600 μg/mL) of HNTs and HNTs-FITC-ICG-RBCM suspensions were added in the wells and incubated at 37 °C for 24 h, respectively. Afterward, CCK-8 assay was performed to study the cell viability of MCF-7 cells and HUVCEs in the presence of HNTs and HNTs-FITC-ICG-RBCM according to the previous paper [30]. In addition, to visualize the viability of MCF-7 cells and HUVECs after treatment with different concentrations of HNTs-FITC-ICG-RBCM, 10 μL of AO/EB dual-fluorescent dyes were added to stain the cells. Subsequently, the cells were observed using a fluorescent microscope (EVOS FL Cell Imaging System). The PBS group without HNTs-FITC-ICG-RBCM was set as the control group.

### 2.9. In Vitro Phototherapy Effect

To study the effects of NIR light-assisted HNTs-FITC-ICG-RBCM heating on cell death and viability. MCF-7 cells were seeded in 96 well plates (1 × 10^4^ cells/well) and incubated with HNTs-FITC-ICG-RBCM at concentrations of 200, 400 and 600 μg/mL for 24 h, respectively. After incubation, the cells were washed by PBS gently for 5 times to remove the nutrient solution, reducing the influence of nutrient solution on photothermal effect (the nutrient solution would also absorb part of the laser energy). Then, the cleaned cells were irradiated using 808 nm laser at a power of 1.5 W/cm^2^ for 2, 4, 6 and 8 min, respectively. The cells were cultured for two hours after irradiation and CCK-8 assay was performed subsequently to examine the cell viability. Briefly, the cells in wells were mixed with 10 μL of CCK8 reagent and incubated for 4 h. The supernatant was obtained by centrifugation at 15,000 rpm for 5 min and the absorbance was detected by a microplate reader (Spectra Max M5, Bio-Tek Instruments, Inc., Winooski, VT, USA) at 450 nm. 

In addition, CCK-8 assay, AO/EB staining assay and cell apoptosis assay were implemented to evaluate the phototoxicity of HNTs-FITC-ICG-RBCM-SA-EpCAM. 400 μg/mL of HNTs and HNTs-FITC-ICG-RBCM-SA-EpCAM were used to treat MCF-7 cells in 96 well plates for 24 h, and cells treated with PBS were used as control in the meanwhile. PBS was applied to wash the cells in well for 5 times. Then, the cells were irradiated with 808 nm laser at a power of 1.5 W/cm^2^ for 8 min. The CCK-8 assay was exerted to measure the cell viability, steps as described above. Afterward, AO/EB staining assay was performed to visualize the death and viability of MCF-7 cells. The AO/EB dual-fluorescent dyes were used to stain the irradiated MCF-7 cells in 96 well plates. The cells were then washed by PBS for 3 times and observed with fluorescent microscope.

In cell apoptosis assay, the cell death was examined by Annexin V-FITC/PI apoptosis detection kit. MCF-7 cells were seeded in 12-well plates at the density of 5 × 10^4^ cells per well and cultured for 24 h. Then, HNTs and HNTs-FITC-ICG-RBCM-SA-EpCAM of a concentration of 400 μg/mL were added into the wells while PBS was also employed to treat the MCF-7 cells as a control. After incubation in three different media for 24 h, the cells were irradiated by 808 nm laser at the power of 1.5 W/cm^2^ for 8 min. The cells were then washed with PBS for 3 times, trypsinized and collected by centrifugation, and dispersed in 0.5 mL of Annexin binding buffer subsequently. Annexin V-FITC and propidium iodide (PI) were applied to stain the cells following the operating instructions. After staining, the cells were detected by flow cytometry. 

### 2.10. Cellar Uptake

Confocal laser scanning microscopy (CLSM) was employed to detect the cellar uptake of HNTs-FITC-ICG-RBCM-SA-EpCAM. MCF-7 cells were incubated in Petri dishes at 37 °C under 5% CO_2_ for 24 h. Then, the cells were treated with HNTs-FITC-ICG-RBCM-SA-EpCAM (400 μg/mL) for 2, 4 and 6 h, respectively. The culture media was removed, and the MCF-7 cells were washed by PBS for 3 times and fixed by paraformaldehyde solution (4%) for 20 min. Finally, the cellar uptake of HNTs-FITC-ICG-RBCM-SA-EpCAM was observed using CLSM (LSM880, Carl Zeiss AG, Oberkochen, Germany).

## 3. Results and Discussion

### 3.1. Characterization of HNTs-FITC-ICG-RBCM

**Figure 1:**
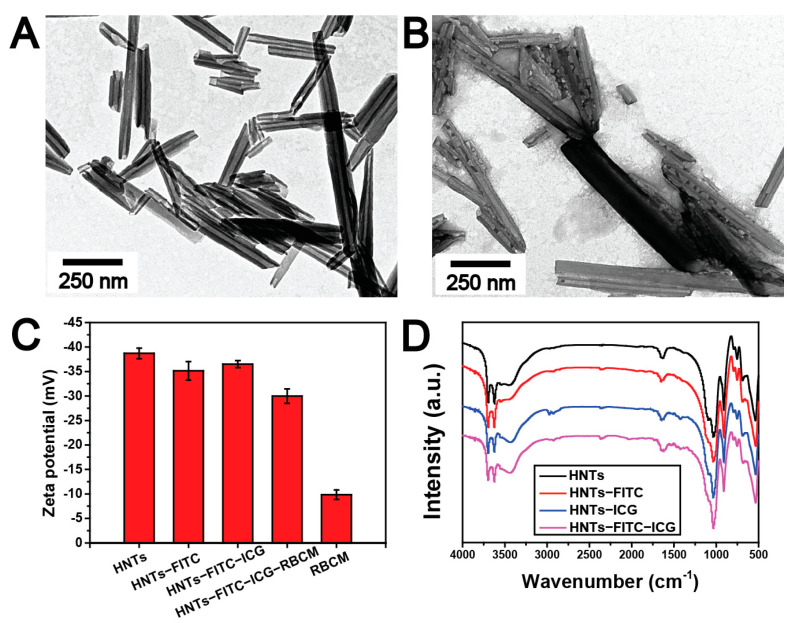
TEM images of HNTs (**A**) and HNTs-FITC-ICG-RBCM (**B**), zeta potential (**C**) of HNTs, HNTs-FITC, HNTs-FITC-ICG, HNTs-FITC-ICG-RBCM and RBCM, FTIR spectrum (**D**) of HNTs, HNTs-FITC, HNTs-ICG and HNTs-FITC-ICG.

The morphology and physicochemical property of the prepared HNTs-FITC-ICG-RBCM were firstly characterized. TEM images of raw HNTs and HNTs-FITC-ICG-RBCM are presented in Figure 1. As showed in Figure 1A, bulk of the raw HNTs have an approximate length varying from 100 nm to 500 nm, which exhibit typical tubular structures with hollow lumen. After loading with FITC and ICG, the lumen of HNTs was filled with ICG through the electrostatic interaction in Figure 1B. Moreover, we observe that HNTs-FITC-ICG are surrounded by a layer of gray matter, indicating the successful encapsulation of red blood cell membrane on HNTs. It should be noticed that broad distribution of length and size of the nanotubes may influence the biological distribution and pharmacokinetics to some extent. Ultrasonic scission can be employed to prepare homogeneous and length controllable HNTs for drug delivery application [42]. 

From the zeta potentials of HNTs-FITC-ICG-RBCM and each intermediate, the surface property change of HNTs was evident (Figure 1C). HNTs-FITC were negatively charged with −35.1 mV. After interacting with ICG, the zeta potentials slightly decreased (−36.5 mV), indicating the negatively charged ICG entered the HNTs lumen and neutralized the positive charges within the lumen [43]. However, after being wrapped by the cell membrane, the zeta potentials changed from −36.5 mV to −30.0 mV. The increase of HNTs potential is probably due to electrostatic shielding of HNTs by cell membrane. 

The FTIR spectral measurement was applied to further explore the structure of HNTs-FITC-ICG (Figure 1D). Raw HNTs’ FTIR spectrum showed the peaks at 907 cm^−1^ and 1028 cm^−1^ attributed to the O–H deformation of the aluminum hydroxyl groups and Si–O stretching, respectively, which is consistent with the previous report [44]. The presence of FITC on HNTs is demonstrated by the peak at 2930 cm^−1^ in the FTIR spectrum, which originated from the C–H stretching from the benzene ring. For HNTs-ICG, the peak at 1424 cm^−1^ was attributed to CH_3_ asymmetric bending of ICG [45]. It could be found that the peaks at 2930 cm^−1^ and 1424 cm^−1^ were also observed in the spectrum of HNTs-FITC-ICG, which suggested the successful incorporation of FITC and ICG with HNTs.

### 3.2. Photothermal Effect and Stability of HNTs-FITC-ICG

**Figure 2:**
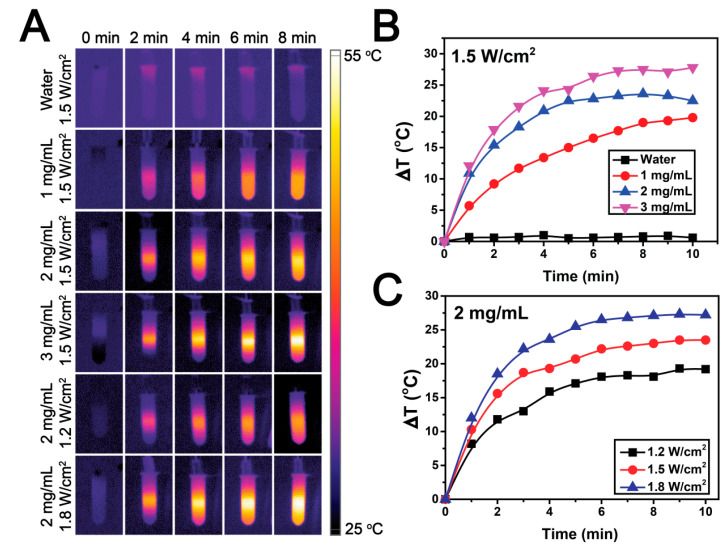
Temperature image of HNTs-FITC-ICG suspension at different concentration and under different power of 808 nm laser (**A**), temperature curve of 1 mL HNTs-FITC-ICG suspension at different concentrations and under 1.5 W/cm^2^ of 808 nm laser (**B**), temperature curve of 1 mL HNTs-FITC-ICG suspension at concentration of 2 mg/mL and under different power of 808 nm laser (**C**).

To evaluate the photothermal effect of HNTs-FITC-ICG, different concentrations of HNTs-FITC-ICG suspension were irradiated under different power of 808 nm laser, and the thermal images were recorded using an infrared thermal imaging camera every 2 min. Obviously, different from the constant temperature of the control group (pure water), the temperature of HNTs-FITC-ICG suspension increased over time (from dark purple to bright yellow) in Figure 2A, indicating the apparent photothermal effect of HNTs-FITC-ICG. The phenomenon of temperature distribution in the tubes is related to the limited laser penetration. In this work, 808 nm laser was used to irradiate the eppendorf tubes containing HNTs-FITC-ICG suspensions from the top down. As the laser penetrated down from the upper layer of the suspension, its energy was continuously weakened due to the absorption of the upper layer of suspension, which resulted in a lower temperature of the lowermost suspension. Meanwhile, due to the partial settlement of the HNTs samples over time, there were more HNTs-FITC-ICG in the middle of the eppendorf tubes, presenting the highest temperature. In addition, the photothermal efficiency of HNTs-FITC-ICG solutions with various concentrations were investigated under the 808 nm laser with constant irradiation intensity of 1.5 W/cm^2^ (Figure 2B). The maximum temperature changes were 19.8 °C, 23.6 °C and 27.8 °C for HNTs-FITC-ICG concentrations under 1 mg/mL, 2 mg/mL and 3 mg/mL, respectively. The maximum temperature change of 27.8 °C was achieved in 3 mg/L of HNTs-FITC-ICG suspension, which indicated that the photothermal efficiency increased with the concentration of HNTs-FITC-ICG. Similarly, as shown in Figure 2C, when the HNTs-FITC-ICG suspension with fixed concentration of 2 mg/mL was exposed to 808 nm laser of different power, the temperature change of HNTs-FITC-ICG suspension increased with the increase of laser power. These results suggested that the photothermal effect is positively correlated with both the concentration of HNTs-FITC-ICG and the power of 808 nm laser. 

**Figure 3:**
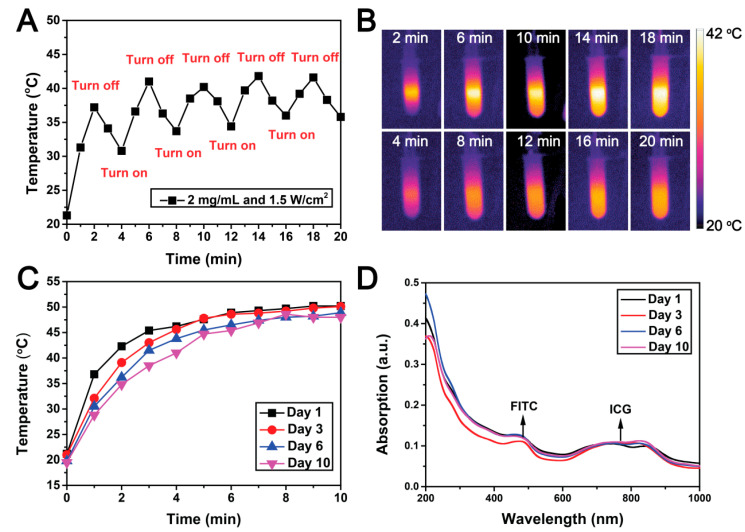
Temperature curve (**A**) and image (**B**) of 1 mL HNTs-FITC-ICG suspension at concentration of 2 mg/mL and under 1.5 W/cm^2^ of 808 nm laser, temperature curve (**C**) and UV-vis spectrum (**D**) of HNTs-FITC-ICG suspension tested in day 1, day 3, day 6 and day 10.

We also performed the photothermal stability experiment of HNTs-FITC-ICG. As shown in Figure 3A,B, the temperature changes of HNTs-FITC-ICG suspension after irradiation merely exhibited slight changes among five repeated cycles of laser irradiation. Furthermore, the photothermal properties of HNTs-FITC-ICG did not become worse because of repeated cyclic irradiation, demonstrating the remarkable photothermal stability of the nanoparticles. Moreover, the time stability of HNTs-FITC-ICG suspension was studied from two aspects of photothermal effect and structural stability over a period of 10 days. Compared with the other three groups, the maximum temperature of HNTs-FITC-ICG suspension after irradiation for 10 min of day 10 group reached 48 °C, only 2.2 °C lower than day 1 group, which confirmed the time-invariant photothermal effect of HNTs-FITC-ICG. The UV-vis spectrum (Figure 3D) shown that the position and intensity of the absorption peaks of FITC and ICG kept nearly unchanged over time, which suggested the structure of HNTs-FITC-ICG was almost unchanged, further indicating the excellent time stability of it.

### 3.3. In Vitro Hemocompatibility of HNTs-FITC-ICG-RBCM

**Figure 4:**
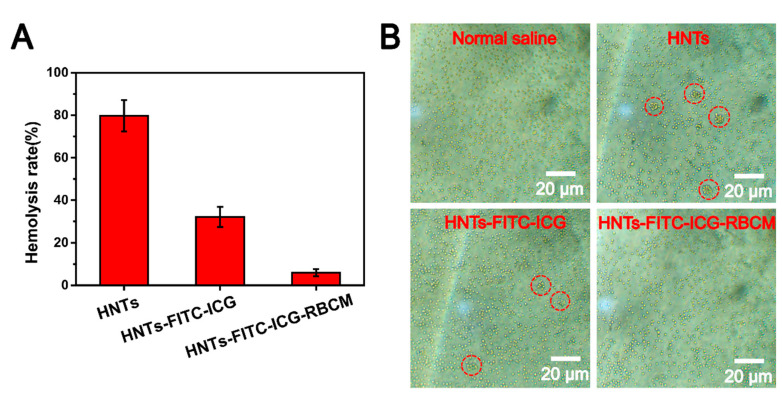
Hemolysis rate (**A**) of HNTs, HNTs-FITC-ICG and HNTs-FITC-ICG-RBCM and red blood cell images (**B**) interacted with different particles for 24 h. The red circles represent the aggregated red blood cells.

Hemocompatibility is a significant index to judge the feasibility of biomedical materials in direct contact with blood. Thus, the hemolytic assay of HNTs-FITC-ICG-RBCM is necessary, and the hemolysis rate results of HNTs-FITC-ICG-RBCM are presented in Figure 4A. It has been proved that hemolysis of nanoparticle is related to porosity, geometry and surface groups [46]. It can be seen that the raw HNTs exhibited a high hemolysis rate of 79.7%, which was consistent with our previous studies that raw HNTs possessed poor hemocompatibility [30]. However, other previous studies showed a contrary result regarding the hemolysis rate of raw HNTs [47]. That is because surface geometry of HNTs depends on its original area which significantly affects the hemolysis. As can be seen from the TEM images (Figure 1A), the sharp edge structure of our HNTs is the primary cause of the high hemolysis rate. However, after the loading of ICG and encapsulation by RBCM, the hemolysis rate of HNTs-FITC-ICG-RBCM decreased to 5.9%. The result suggested that the encapsulation of RBCM reduced the sharpness of HNTs edge and effectively improved the blood compatibility of the nanoparticles. To further illustrate the interactions between the particles and the red blood cells, the red blood cells were treated with HNTs, HNTs-FITC-ICG and HNTs-FITC-ICG-RBCM suspensions for 24 h, respectively, and 0.9% normal saline was used as negative control. The microscope images (Figure 4B) shown that the red blood cells exhibited great dispersity and possessed normal cell morphology in HNTs-FITC-ICG-RBCM media, similar to the negative control group. However, red blood cells showed a degree of aggregation in HNTs and HNTs-FITC-ICG media, which was related to the coagulation effect of HNTs. These results demonstrated that HNTs-FITC-ICG-RBCM possessed good hemocompatibility and could be recommended to be used as blood-contact medicine for various therapeutic applications.

### 3.4. In Vitro Cytotoxicity of HNTs-FITC-ICG-RBCM

**Figure 5:**
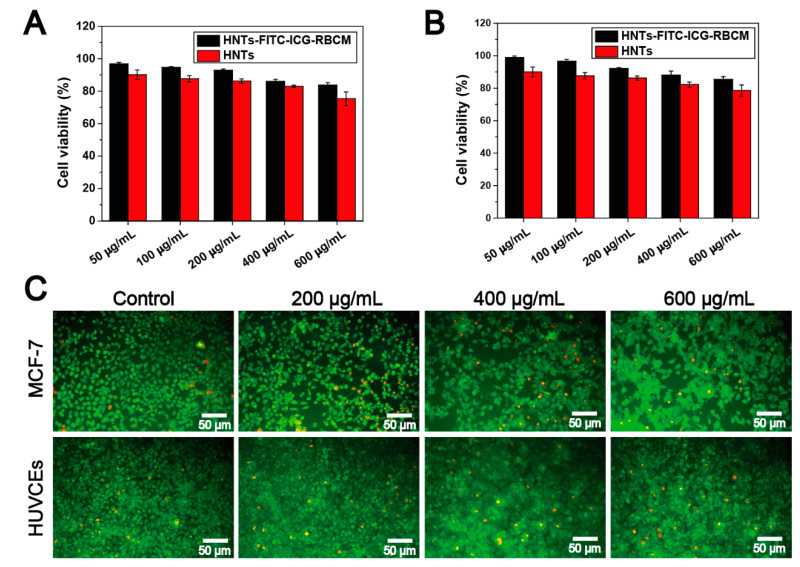
Relative cell viability (**A**): MCF-7, (**B**): HUVCEs and AO/EB image (**C**) of MCF-7 and HUVCEs cultured with HNTs or HNTs-FITC-ICG-RBCM at different concentrations for 24 h.

To evaluate the cytotoxicity of HNTs and HNTs-FITC-ICG-RBCM, CCK-8 assay and AO/EB assay were exerted to examine the cell viability using the MCF-7 cells and HUVCEs cells which co-cultured with different concentrations of the nanoparticles for 24 h. As shown in Figure 5A,B, MCF-7 cells and HUVCEs cells showed high cell viability (over 90%) after culturing in HNTs-FITC-ICG-RBCM suspension with the concentration below 200 μg/mL, indicating the low cytotoxicity of HNTs-FITC-ICG-RBCM. However, the cell viability was lower than 90% when the concentration was higher than 50 μg/mL in HNTs medium. The result of CCK-8 assay suggested that the functionalization of ICG and RBCM lowered the cytotoxicity of the nanotubes, which endowed HNTs satisfied biocompatibility. In addition, from the AO/EB staining images (Figure 5C), the MCF-7 cells treated with HNTs-FITC-ICG-RBCM only produced a little more death of cells (red fluorescence) than the control group. Furthermore, the apoptosis rate of HUVCEs cells treated with different concentrations of HNTs-FITC-ICG-RBCM was nearly the same as that of control group. Thus, in vitro cytotoxicity study demonstrated that HNTs-FITC-ICG-RBCM was highly biocompatible and could be used in vivo applications.

### 3.5. In Vitro Photocytotoxicity of HNTs-FITC-ICG-RBCM-SA-EpCAM

Due to the unique needle-like morphology and high aspect ratio, carbon nanotubes are considered to have the ability to penetrate cell membranes directly through energy-independent mechanisms [48]. Similarly, HNTs with cylindrical shape and high aspect ratio can be ingested into cells by the same mechanism [49]. The presence of anti-EpCAM allowed selective cellular uptake of HNTs-FITC-ICG-RBCM-SA-EpCAM by MCF-7 cells. Under laser irradiation, ICG loaded in the HNTs lumen yield detrimental reactive oxygen species and thermal effects after absorbing near-infrared light, leading to irreversible cells damage and inducing cells apoptosis [50].

**Figure 6:**
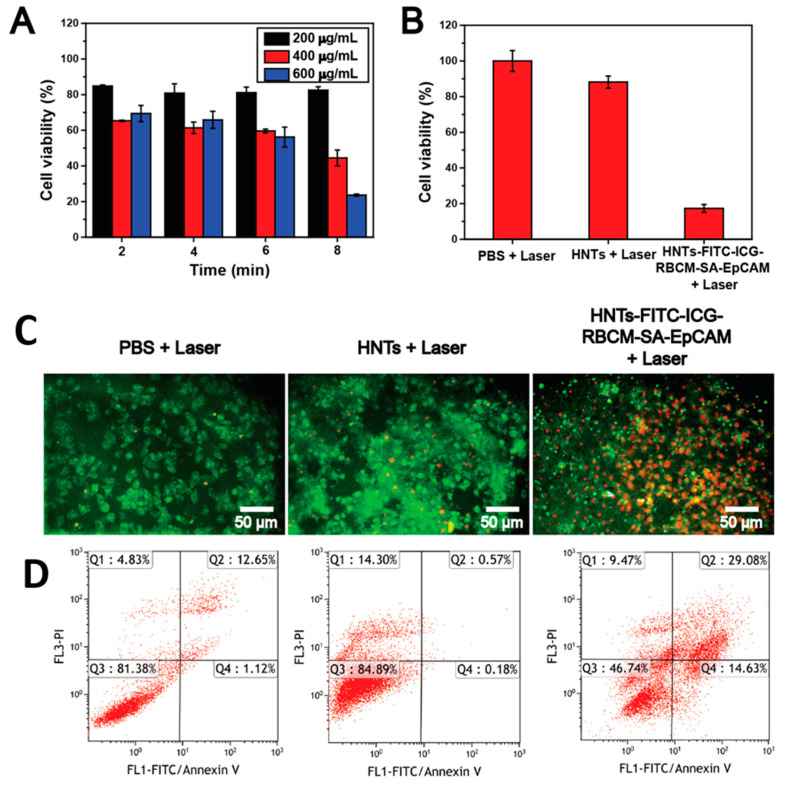
Cell viability (**A**) of MCF-7 cells cultured with different concentrations of HNTs-FITC-ICG-RBCM by irradiation with 808 nm laser at 1.5 W/cm^2^; cell viability (**B**), AO/EB staining photo (**C**) and ratio of apoptosis (**D**) of MCF-7 cultured with PBS, HNTs and HNTs-FITC-ICG-RBCM-SA-EpCAM (concentration: 400 μg/mL).

In our work, the photocytotoxicity of HNTs-FITC-ICG-RBCM was first evaluated by CCK-8 assay with MCF-7 cells. The MCF-7 cells were cultured with different concentrations of HNTs-FITC-ICG-RBCM solutions under 808 nm laser at 1.5 W/cm^2^ for different intervals, and then the cell viability of MCF-7 cells was examined. As shown in Figure 6A, when the concentration of HNTs-FITC-ICG-RBCM was 200 μg/mL, the cell viability of MCF-7 cells remained above 80% and barely changed over irradiation time; whereas, when the concentration of HNTs-FITC-ICG-RBCM increased, cell viability decreased gradually with the prolonging of irradiation time. It was found that the survival rate of cells was only 23.6% at high concentrations of HNTs-FITC-ICG-RBCM in 600 μg/mL after being irradiated for 8 min. The results indicated that the cytotoxicity of HNTs-FITC-ICG-RBCM was concentration and irradiation time dependent. To investigate the photocytotoxicity of HNTs-FITC-ICG-RBCM-SA-EpCAM, the CCK-8 assay, AO/EB staining and cell apoptosis assay were performed. For all the assays, MCF-7 cells were incubated in 400 μg/mL of HNTs and HNTs-FITC-ICG-RBCM-SA-EpCAM dispersion, and the power of laser was 1.5 W/cm^2^ with the irradiation time of 8 min, using the PBS solution as the control group. The result of CCK-8 assay was shown in Figure 6B that the PBS and HNT groups exhibited a low photocytotoxicity of 100% and 88.2% cell viability, respectively. On the contrary, the HNTs-FITC-ICG-RBCM-SA-EpCAM showed a low cell viability of 17.3%, reflecting the outstanding photocytotoxicity of HNTs-FITC-ICG-RBCM-SA-EpCAM. It was clear that MCF-7 cells treated with HNTs-FITC-ICG-RBCM-SA-EpCAM under the same conditions shown a lower cell viability than those treated with HNTs-FITC-ICG-RBCM (44.4%). As a specific recognition molecule, anti-EpCAM endowed HNTs-FITC-ICG-RBCM-SA-EpCAM the ability to efficiently targeting cancer cells through molecular recognition, which greatly improved the anti-cancer efficiency of the nanoparticles. Meanwhile, a mass of red cell in AO/EB images (Figure 6C) at HNTs-FITC-ICG-RBCM-SA-EpCAM group demonstrated the high cell apoptosis rate under laser. In addition, as shown in Figure 6D, the results of the flow cytometry apoptosis analysis were consistent with the results of AO/EB assay, resulting in the highest cell apoptosis (Q2 + Q4) of 43.71% in MCF-7 cells for HNTs-FITC-ICG-RBCM-SA-EpCAM group. AO/EB staining data and FACS data on cell apoptosis after laser irradiation showed slightly different since the principles and sampling time of these two methods were different. To sum up, comparing to the raw HNTs, HNTs-FITC-ICG-RBCM-SA-EpCAM exhibited high photocytotoxicity, demonstrating their in vivo phototherapy potential.

### 3.6. Cellular Uptake

**Figure 7:**
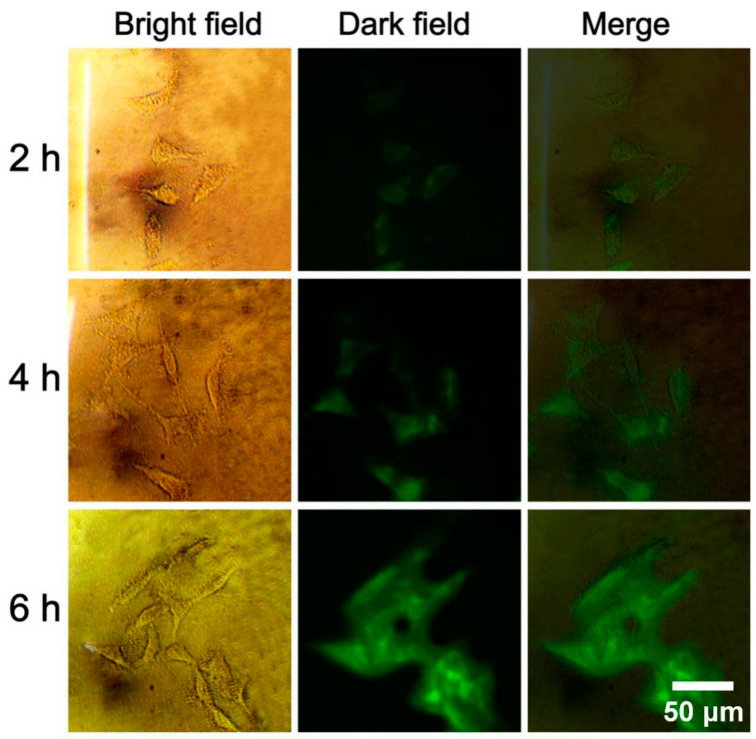
Fluorescent photograph of MCF-7 treated with HNTs-FITC-ICG-RBCM-SA-EpCAM at different time intervals.

The intracellular uptake of HNTs-FITC-ICG-RBCM-SA-EpCAM in MCF-7 cells was investigated via the green fluorescence of FITC, since the sterilization process of HNTs-FITC does not affect the fluorescence of FITC. As displayed in Figure 7, the confocal microscopy images showed the fluorescent pictures of MCF-7 cells incubated with HNTs-FITC-ICG-RBCM-SA-EpCAM at different time periods. It could be observed that prominent green fluorescence at the interior of the MCF-7 cells, indicating the effective cellular uptake of HNTs-FITC-ICG-RBCM-SA-EpCAM. The results proved that the grafted anti-EpCAM could induce the uptake of HNTs-FITC-ICG-RBCM-SA-EpCAM in cell effectively. Moreover, the fluorescence intensity enhanced with the increase of co-culture time, which suggested that cells absorbed more HNTs-FITC-ICG-RBCM-SA-EpCAM over time. Therefore, it is expected that the modified nanotubes with photothermal and targeting properties can enter breast cancer cells effective in further in vivo cancer therapy. 

## 4. Conclusions

In summary, a novel HNTs-based nanocarrier has been designed for effective targeting and phototherapy of breast cancer. Fluorescence nanoparticles HNTs-FITC-ICG with photothermal effects were successfully prepared by loading ICG into the lumen of FITC-labeled HNTs. The nanoparticles possessed good photothermal conversion ability, and the maximum temperature change of 27.8 °C was achieved at 1.5 W/cm^2^ laser power. The encapsulation of HNTs can effectively improve the stability of ICG. In addition, the wrapping of RBCM strongly improved the biocompatibility of HNTs-FITC-ICG-RBCM, which endowed the nanoparticles low cytotoxicity and hemolysis rate in cell experiments. The conjugation of anti-EpCAM promoted the selective accumulation of HNTs-FITC-ICG-RBCM-SA-EpCAM in the tumor region. The results showed that the novel nanoparticles owned the ability to target into cancer cells and show high anticancer efficiency under NIR laser irradiation. Hence, HNTs-FITC-ICG-RBCM-SA-EpCAM has potential application in the treatment of breast cancer.

## Data Availability

Data present in this study are available on request from the corresponding author.

## Sample Availability

Not available.
